# Hereditary hemorrhagic telangiectasia patient with pulmonary arteriovenous malformation: A case report

**DOI:** 10.1097/MD.0000000000047435

**Published:** 2026-01-30

**Authors:** Ching Wen Pang

**Affiliations:** a Department of Nursing, National Taiwan University Hospital, Taipei City, Taiwan (R.O.C.).

**Keywords:** arteriovenous malformation, ENG gene mutation, hereditary hemorrhagic telangiectasia, Osler–Weber–Rendu disease, thalidomide

## Abstract

**Rationale:**

Hereditary hemorrhagic telangiectasia (HHT) is a rare autosomal dominant vascular disorder characterized by recurrent epistaxis, mucocutaneous telangiectasias, and visceral arteriovenous malformations (AVMs), which can lead to serious bleeding and organ complications. Early diagnosis and multidisciplinary management are essential.

**Patient concerns:**

A 61-year-old man presented with intermittent melena, hematochezia, and severe epistaxis. While undergoing hemodialysis for newly diagnosed end-stage renal disease, he developed respiratory distress, hemoptysis, and hemorrhagic shock.

**Diagnoses:**

Clinical suspicion of HHT was supported by imaging that revealed a pulmonary AVM. Genetic testing confirmed a heterozygous endoglin mutation, establishing a diagnosis of HHT type 1.

**Interventions:**

He underwent endoscopic hemostasis, tracheostomy, pulmonary AVM embolization, inhaled tranexamic acid, and thalidomide to control bleeding.

**Outcomes:**

Thalidomide reduced the frequency of transfusions and the severity of bleeding; however, intermittent bleeding persisted. The patient remained ventilator-dependent due to respiratory compromise.

**Lessons:**

HHT should be suspected in patients presenting with recurrent bleeding at multiple sites. Genetic testing is essential for confirming the diagnosis, while a combination of endoscopic, interventional, and antiangiogenic therapies plays a crucial role in management. Despite these treatments, controlling bleeding remains challenging, highlighting the importance of individualized, multidisciplinary care.

## 1. Introduction

Rendu–Osler–Weber syndrome, also known as hereditary hemorrhagic telangiectasia (HHT), is an autosomal dominant genetic disorder with an estimated prevalence of 1 in 5000, with an even lower prevalence in Asian populations.^[1]^ The clinical diagnosis of HHT is based on the Curaçao criteria, established by the Scientific Advisory Board of the HHT Foundation International, which include: epistaxis—spontaneous and recurrent nosebleeds; telangiectasias—localized dilated vessels on the lips, oral cavity, fingers, and nose; visceral lesions—such as gastrointestinal telangiectasias (with or without bleeding) and arteriovenous malformations (AVMs) in the stomach, liver, brain, lungs, and spinal cord; and family history—a close relative diagnosed with HHT. A definitive diagnosis is made when 3 criteria are met, while 2 criteria indicate a suspected case.^[2]^

To date, pathogenic mutations have been identified in one of the following genes in 97% of patients with a definitive clinical diagnosis of HHT, categorizing the condition into 3 subtypes. Type 1 HHT is caused by mutations in the endoglin gene, while type 2 HHT results from mutations in the activin A receptor-like type 1 gene. The third subtype, known as juvenile polyposis-HHT overlap syndrome, is associated with mutations in the Mothers against decapentaplegic homolog 4 gene. Additionally, several other gene mutations have yet to be definitively linked to HHT.^[3]^

Approximately 90% of patients experience recurrent epistaxis and telangiectasias. Other symptoms include gastrointestinal bleeding (25%–30%), which can result in melena and severe microcytic anemia requiring frequent blood transfusions; pulmonary arteriovenous malformations (50%), which may cause dyspnea, hemoptysis, and paradoxical embolism; cerebral vascular malformations (10%), which can lead to headaches, seizures, and focal neurological deficits; and hepatic arteriovenous malformations (40%–70%), which are often asymptomatic but may present with signs of high-output cardiac failure and liver decompensation, ultimately necessitating liver transplantation.^[4]^

According to literature reviews, pathogenic mutations associated with HHT have been shown to increase levels of vascular endothelial growth factor (VEGF) and transforming growth factor-beta, leading to abnormal vascular proliferation and subsequent bleeding. For treatment, thalidomide, an antiangiogenic drug, is recommended. Thalidomide works by blocking VEGF receptors, thereby strengthening blood vessels in patients with HHT, reducing the frequency of epistaxis, improving anemia, and decreasing the need for transfusions. However, the drug is associated with side effects, including drowsiness, peripheral neuropathy, heart failure, and thrombosis, which limit its suitability for long-term use. Nonetheless, it shows significant potential for managing HHT-related symptoms.^[5,6]^

## 2. Patient information

A 61-year-old male initially presented in April 2024 with intermittent episodes of melena and hematochezia. His past medical history was unremarkable at that time. However, in July 2024, he was diagnosed with end-stage renal disease and began regular hemodialysis through a Hickman catheter placed in the right subclavian vein.

## 3. Clinical findings

One week after initiating hemodialysis, the patient developed acute chest tightness, dyspnea, and diaphoresis. Clinical suspicion of non-ST-elevation myocardial infarction prompted the initiation of dual antiplatelet therapy, including aspirin, clopidogrel, and heparin. Shortly thereafter, he developed severe epistaxis and gastrointestinal bleeding, necessitating intubation for airway protection and transfer to the intensive care unit, where he received desmopressin acetate therapy to control nasal bleeding.

Despite initial stabilization and extubation, the patient experienced recurrent episodes of epistaxis, hemoptysis, and oxygen desaturation, necessitating re-intubation. In August 2024, he underwent a tracheostomy and electrocauterization. Tranexamic acid inhalation therapy was initiated to manage ongoing bleeding. Subsequent panendoscopy revealed superficial gastritis and gastric erosions, but no active bleeding was detected. Bronchoscopy also failed to identify a source of active bleeding. The patient stabilized on single antiplatelet therapy with Plavix.

## 4. Diagnostic assessment

Approximately 1 week later, the patient experienced hemorrhagic shock, with hemoglobin levels dropping from 9.2 to 5.5 g/dL. Emergency management included a massive blood transfusion and a computed tomography scan of the chest, abdomen, and pelvis to identify a potential bleeding source. Although no active hemorrhage was detected, a pulmonary AVM was identified in the right lower lobe. Persistent episodes of epistaxis and cutaneous bleeding raised a strong clinical suspicion of HHT.

To confirm the diagnosis, next-generation whole-exome sequencing was performed at the patient’s expense. This analysis revealed a pathogenic heterozygous single-nucleotide duplication in the endoglin gene (c.958dup, p.Ala320GlyfsTer14), confirming a diagnosis of HHT type 1 in September 2024.

## 5. Therapeutic intervention

**Figure 1.**
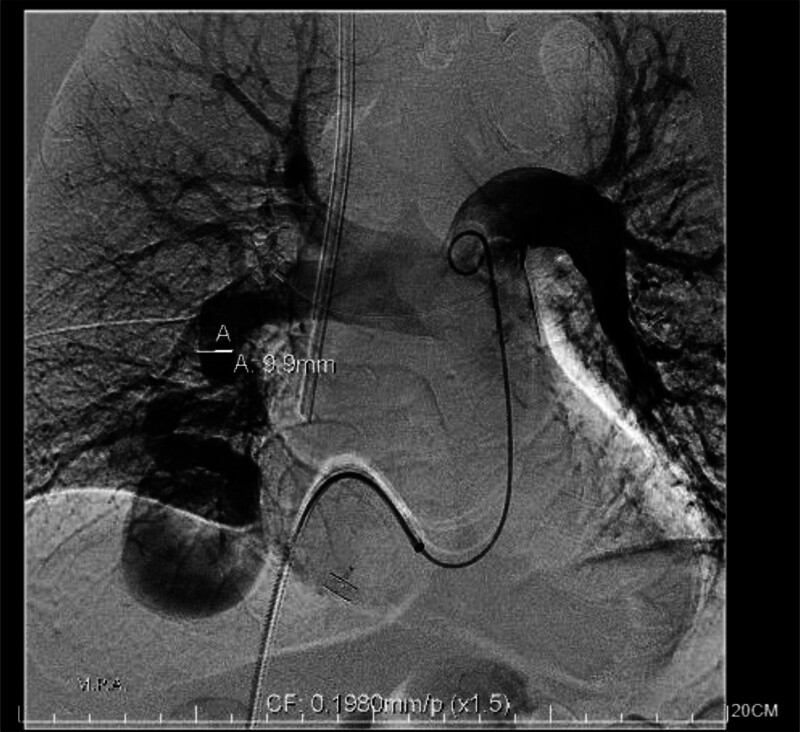
A pulmonary arteriovenous malformation (AVM) in the right lower lobe.

During a subsequent hemodialysis session, the patient experienced a seizure and oxygen desaturation, necessitating the initiation of daily levetiracetam (1000 mg) and mechanical ventilation. High-output cardiac failure secondary to the pulmonary AVM was suspected. On September 30, 2024, the patient underwent successful transarterial embolization of the AVM (Fig. 1).

**Figure 2.**
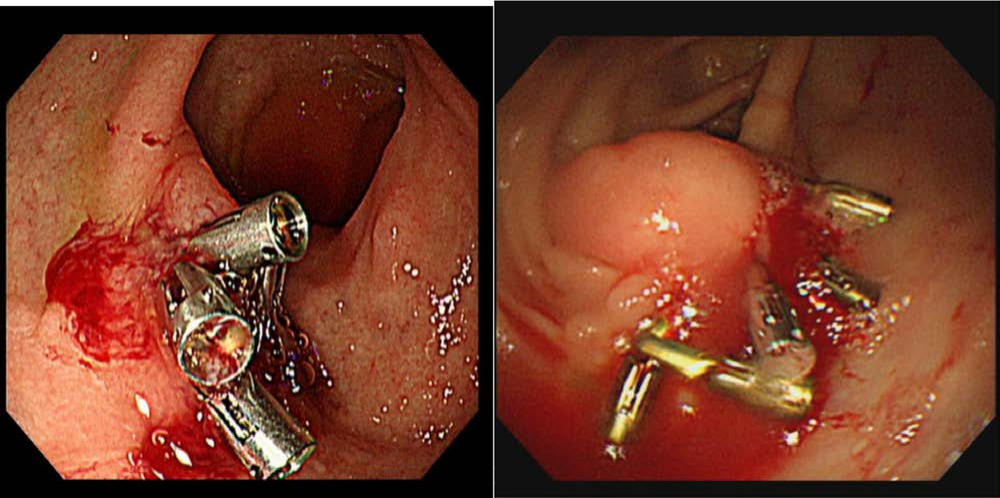
Endoscopic findings in the patient. (A) Small intestine with hemoclip. (B) Large intestine with hemoclip.

Despite this intervention, endoscopic evaluations continued to reveal multiple gastrointestinal vascular malformations located in the jejunum, ileum, and colon. These lesions were treated using argon plasma coagulation (APC) and hemoclipping (Fig. 2); however, these measures did not significantly reduce the frequency of transfusions. In October 2024, thalidomide therapy was initiated. Under thalidomide treatment, the frequency of transfusions markedly decreased, although the patient continued to experience occasional melena and hemoptysis related to recurrent vascular malformations.

## 6. Follow-up and outcomes

The patient responded well to thalidomide, experiencing no major adverse effects during the treatment period. Although bleeding episodes persisted, their frequency and severity were significantly reduced, resulting in decreased dependence on transfusions and improved symptom management. A summary of the patient’s clinical course is presented in Table 1.

**Table 1:** Summary of the patient’s clinical course, diagnostic evaluations, and therapeutic interventions.

Date	Event description
April 2024	Presented with intermittent melena and hematochezia.
July 2024	Diagnosed with ESRD and initiated hemodialysis via right subclavian Hickman catheter.
Mid-July 2024	Developed chest tightness; started on dual antiplatelet therapy following a suspected NSTEMI.
Mid-July 2024	Severe epistaxis and gastrointestinal bleeding; intubated and treated with desmopressin in the ICU.
Late July 2024	Experienced recurrent bleeding; underwent a tracheostomy and was started on inhaled tranexamic acid.
Early August 2024	Endoscopies revealed gastritis without active bleeding.
Mid-August 2024	Hemorrhagic shock; CT scan revealed a right lower lobe pulmonary AVM. Suspected HHT due to recurrent bleeding.
September 2024	Genetic testing confirmed the diagnosis of HHT Type 1 (ENG mutation).
Late September 2024	Embolization of the pulmonary AVM was performed.
October 2024	Gastrointestinal vascular malformations were treated with APC and hemoclips. Thalidomide therapy was initiated, resulting in a reduction of transfusion requirements.
Follow-up	Responded well to thalidomide without major adverse effects during the treatment period. Although bleeding episodes persisted, their frequency and severity were significantly reduced, resulting in decreased dependence on transfusions and improved symptom management.

APC = argon plasma coagulation, AVM = arteriovenous malformation, CT = computed tomography, ENG = endoglin, ESRD = end-stage renal disease, HHT = hereditary hemorrhagic telangiectasia, ICU = intensive care unit, NSTEMI = non-ST-elevation myocardial infarction.

## 7. Discussion

HHT is an autosomal dominant genetic disorder for which no curative treatment currently exists. Consequently, symptomatic management remains the primary therapeutic approach.

For frequent epistaxis, first-line treatments may include direct nasal compression, the use of local medications such as vasoconstrictors, electrocautery at the bleeding site, or the use of hemostatic adjuncts like antifibrinolytics and hemostatic packing materials. Subsequent treatments may involve electrocautery, laser ablation, sphenopalatine artery ligation or embolization, septoplasty, and nasal closure surgery.^[7]^ A study by Ashoke et al suggested that the addition of bevacizumab injections following electrocautery may lead to symptom improvement lasting up to 4 months; however, further studies are needed to confirm these findings.^[8]^

Tranexamic acid is an effective antifibrinolytic agent. In a study by Ori Wand et al, inhaled tranexamic acid was found to be a safe and effective treatment option for patients with significant hemoptysis. It can be used as monotherapy or as adjunctive therapy.^[9]^ The study further indicated that nebulized tranexamic acid is more effective and associated with fewer side effects compared to intravenous administration.^[10]^

Patients with HHT often experience gastrointestinal vascular malformations and telangiectasias, which frequently lead to bleeding. Therefore, interventions such as endoscopy—including esophagogastroduodenoscopy and colonoscopy—are essential for identifying and treating active bleeding sites. Hemoclips can be used to control active bleeding, or in cases without active bleeding but with signs of scarring (e.g., adherent clots, visible vessels, and red or black spots), to prevent further hemorrhage.^[11]^ A study by Jorge Atilio Olmos et al, demonstrated that APC effectively controls active bleeding. The study also found that adjusting the power settings of APC according to the location of the vascular malformation can safely and effectively prevent recurrent gastrointestinal bleeding. However, due to the lack of a control group, further randomized controlled trials are necessary to validate these findings.^[12]^

For comprehensive management, daily administration of 100 mg of thalidomide is commonly used as a treatment option. Thalidomide is an antiangiogenic drug that works by blocking VEGF receptors. It has been shown to increase median hemoglobin levels and reduce transfusion dependency.^[13]^ Additionally, thalidomide directly inhibits endothelial cell proliferation, decreases bleeding frequency in HHT patients, and reduces the need for additional blood transfusions.^[14]^ Common side effects include drowsiness, fatigue, constipation, peripheral neuropathy, heart failure, and thromboembolic events (2.9%).^[15,16]^

Frequent bleeding episodes in patients with HHT not only pose a life-threatening risk but can also severely affect the patient’s quality of life, often necessitating frequent hospitalizations for transfusion therapy and endoscopic interventions. Although several treatment strategies have been discussed above, additional clinical trials are needed to validate these approaches and explore new therapies that may further improve patient outcomes.

## 8. Conclusions

HHT is a chronic, autosomal dominant vascular disorder for which there is currently no cure. This case highlights the challenges of managing recurrent, life-threatening bleeding from multiple organs, necessitating coordinated care involving endoscopy, interventional radiology, antifibrinolytics, and antiangiogenic agents. Inhaled tranexamic acid and thalidomide helped reduce bleeding and transfusion needs, but complete control was not achieved. Given the variable treatment efficacy and limited evidence, individualized management based on disease severity and available resources is essential.

This report also highlights the importance of family screening and early genetic testing, as the patient’s younger sister was subsequently diagnosed with pulmonary arteriovenous malformations and has begun genetic follow-up for preventive care.

There is an urgent need for standardized diagnostic and treatment guidelines. Future research should prioritize early genetic screening for at-risk patients to facilitate timely monitoring and prevention. Multicenter studies and long-term data collection are essential to improving outcomes and quality of life for patients with HHT.

## Author contributions

**Conceptualization:** Ching Wen Pang.

**Data curation:** Ching Wen Pang.

**Formal analysis:** Ching Wen Pang.

**Investigation:** Ching Wen Pang.

**Methodology:** Ching Wen Pang.

**Supervision:** Ching Wen Pang.

**Writing – original draft:** Ching Wen Pang.

**Writing – review & editing:** Ching Wen Pang.
